# Remineralization of Dentinal Lesions Using Biomimetic Agents: A Systematic Review and Meta-Analysis

**DOI:** 10.3390/biomimetics8020159

**Published:** 2023-04-15

**Authors:** Ali Azhar Dawasaz, Rafi Ahmad Togoo, Zuliani Mahmood, Azlina Ahmad, Kannan Thirumulu Ponnuraj

**Affiliations:** 1 Department of Diagnostic Dental Sciences, College of Dentistry, King Khalid University, Abha 62529, Saudi Arabia; 2 School of Dental Sciences, Universiti Sains Malaysia, Kubang Kerian 16150, Kelantan, Malaysia; 3 Department of Pediatric Dentistry and Orthodontic Sciences, College of Dentistry, King Khalid University, Abha 62529, Saudi Arabia; 4 Human Genome Centre, School of Medical Sciences, Universiti Sains Malaysia, Kubang Kerian 16150, Kelantan, Malaysia

**Keywords:** tooth remineralization, dentine, biomimetics, polyacrylic acid, sodium trimetaphosphate

## Abstract

The objective of this article was to systematically provide an up-to-date review on the different methods of remineralizing human dentine using different biomimetic agents. The authors performed a systematic search within PubMed, Scopus, and Web of Science in addition to the grey literature in Google Scholar^®^ using MeSH terms. The PICO question was P: human teeth dentinal sections; I: application of biomimetic remineralizing agents; C: other non-biomimetic approaches; O: extent of remineralization and physical properties of remineralized dentine. The initially identified studies were screened for titles and abstracts. Non-English articles, reviews, animal studies, studies involving the resin–dentine interface, and other irrelevant articles were then excluded. The other remaining full-text articles were retrieved. Bibliographies of the remaining articles were searched for relevant studies that could be included. A total of 4741 articles were found, and finally, 39 full-text articles were incorporated in the current systematic review. From these, twenty-six research studies used non-collagenous protein (NCP) analogs to biomineralize dentine, six studies used bioactive materials derived from natural sources, six studies used zinc hydroxyapatite, and one study used amelogenin peptide to induce hydroxyapatite formation on the surface of demineralized dentine. Additive effects of triclosan and epigenin were assessed when combined with commonly available NCPs. Overall, a moderate risk of bias was observed and, hence, the findings of the included studies could be acceptable. A meta-analysis of some similar studies was performed to assess the depth of remineralization and elastic modulus. Despite having high heterogeneity (I^2^ > 90), all the studies showed a significant improvement in biomimetic remineralization efficacy as compared to the control. All the included studies carried out a functional remineralization assessment and found a 90–98% efficacy in the extent of remineralization while the elastic modulus reached 88.78 ± 8.35 GPa, which is close to natural dentine. It is pertinent to note the limitations of these studies that have been carried out in vitro under controlled settings, which lack the effects of a natural oral environment. To conclude, the authors suggest that the biomimetic remineralization of dentine using NCP analogs, bioactive materials, and natural products carries significant potential in treating dentinal lesions; however, more long-term studies are needed to assess their clinical applications in vivo.

## 1. Introduction

Seventy percent carbonated apatite makes up the dentine that is a collagenous mineralized tissue, and the remainder consists of organic collagen, non-collagenous protein, and water. This 30% of dentine plays a key role in remineralization [1,2]. Dental caries is characterized by a process involving an imbalance in remineralization and demineralization [3]. Untreated dental caries is a global pandemic and early childhood caries was found to be the 10th most common disease among 291 health conditions [4], whereas in permanent teeth, it was the most prevalent condition in all of the Global Burden of Disease 2015 (age-standardized prevalence: 34.1%), affecting 2.5 billion people worldwide (95% UI: 2.4 to 2.7 billion) [5]. The demineralization of dentine along with the loss of tooth structure is also seen in regressive alterations of teeth that may cause hypersensitivity to temperature changes during food intake [6]. Demineralization outweighs remineralization in pathological conditions. The exposure of collagen fibers results when the mineral phase of dentine becomes affected, allowing rapid damage of the whole dentine network such as collagen fibril degradation as well as altered mechanical properties [2]. As enamel contains residual seed mineral crystals, it is less difficult for it to attain remineralization than dentine can [7]. However, developmental conditions affecting surface enamel such as Molar Incisor Hypomineralization (MIH) [8] and other causes of enamel hypoplasia will make patients more susceptible to developing caries in the underlying dentine [9]. Such studies were excluded from the present review. Fan et al. [10] found that remineralization did not occur on acid-etched dentine compared to acid-etched enamel under the similar remineralizing conditions. This was caused by less calcified crystals still present in the acid-etched dentine surface, which exposes the organic matrix (primarily type I collagen) [10]. The remineralization of non-infected carious dentine is necessary under the domain of minimally invasive dentistry during critical pulp therapy [11]. There is an availability of numerous non-invasive or minimally invasive caries treatment methods including dental hygiene education, infiltrative resins, xylitol [12], fluorides, and phosphopeptide compounds [13].

The dentine mineralization process is greatly affected by the presence of extracellular matrix proteins in controlling apatite nucleation and growth. It has been found to contain transient amorphous calcium phosphate (ACP) nanoprecursors [14]. Dentinal remineralization is extremely challenging. The ‘ion’ or ‘classical’ theory of remineralization requires the presence of some hydroxyapatite crystals in partially demineralized dentine [15]. A different approach is ‘biomimetic remineralization’, which attempts to infiltrate liquid-like ACP nanoprecursor particles into the demineralized dentine collagen without relying on seed crystallites using a bottom-up remineralization strategy. This could be considered a viable technique for remineralizing demineralized dentine [16].

Although the most abundant organic component of dentine is collagen fibrils, glycoproteins and non-collagenous proteins (NCPs) account for below 10% of the aggregate content of organic components. They take part in crucial roles in the regulation of mineralization [17]. Based on the current understanding of the dental biomineralization process, new avenues have been explored in developing and synthesizing NCP analogs, which are crucial in initiating nucleation and growth of hydroxyapatite crystals in dental hard tissues [18]. The amphiphilic properties of NCPs where the polar groups bind to inorganic ions and the non-polar sidechains governing matrix-to-matrix interactions have been designed [19]. The ease of designing specific motifs and their biocompatibility have led to the harnessing of peptides in remineralization strategies to engineer particular properties [20].

It is fascinating to study the effects of biomimetic agents on human dentine. Only one systematic review [16] has been reported in the literature dated 2015. Thus, an updated information gap exists to this day about the standard protocol in the treatment of dentinal lesions using biomimetic agents. This systematic review’s main goal is to summarize and assess the findings from research projects that show how effective biomimetically active remineralizing agents are in treating dentinal lesions.

The objective of this article is to systematically review various published articles on biomimetic remineralizing agents on human dentine including different types of NCPs and natural products, and to assess the outcome measures in the included studies using quantitative meta-analysis.

## 2. Materials and Methods

### 2.1. Question under Review

This was set as “What is the effectiveness and mode of action of different biomimetic remineralizing agents in lesions affecting human dentine?” after registering the review protocol with PROSPERO (https://www.crd.york.ac.uk/PROSPERO (accessed on 25 January 2023)) bearing registration number CRD42023386859.

### 2.2. Search Strategy

Databases including PubMed, Scopus, and Web of Science together with sources of the grey literature were explored up to November 2022. For the search in Web of Science and Scopus, the applied terms were Biomimetic AND Remineral* agent* AND humans. For the PubMed database, the search was carried out using combinations of keywords in the title and abstract and MeSH terms: ((TI = (remineral*)) AND TI = (dentin* caries)).

Google Scholar^®^ was used to conduct a grey literature search to finish the screening process and an individual journal search in the genre(s) of Nanomaterials and biochemistry-related journals. Review articles served as additional references for the search process [16,21]. Accessibility for the full text was then checked for all the included studies.

The relevant articles were then assessed based on the following criteria:

Inclusion:

- In vitro studies;
- Randomized Controlled Trials or Case series;
- Retrospective or prospective Cohort;
- Studies using human teeth or in vivo human trials.

Exclusion:

- Case reports;
- Narrative/systematic reviews;
- Non-English language articles;
- Animal studies;
- Human trials that lacked information;
- Studies assessing remineralization of hypoplastic lesions affecting enamel/dentine.

### 2.3. Criteria for Selection

The Preferred Reporting Items for Systematic Review and Meta-analysis (PRISMA) flow diagram 2020 was employed to implement a systematic methodology [22]. The search primarily focused on recording the prevailing literature on the biomimetically induced remineralization of human teeth using different agents. Then, the search was restricted to consist of articles that made use of these agents on lesions affecting dentine. The amount of the remineralization of the dentinal tubules was the main outcome measure evaluated. The search span was until November 2022.

The PICO [23] statement in this review process consisted of the following factors: P: human teeth dentinal sections; I: application of biomimetic remineralizing agents; C: other non-biomimetic approaches; O: extent of remineralization and physical properties of remineralized dentine.

**Figure 1:**
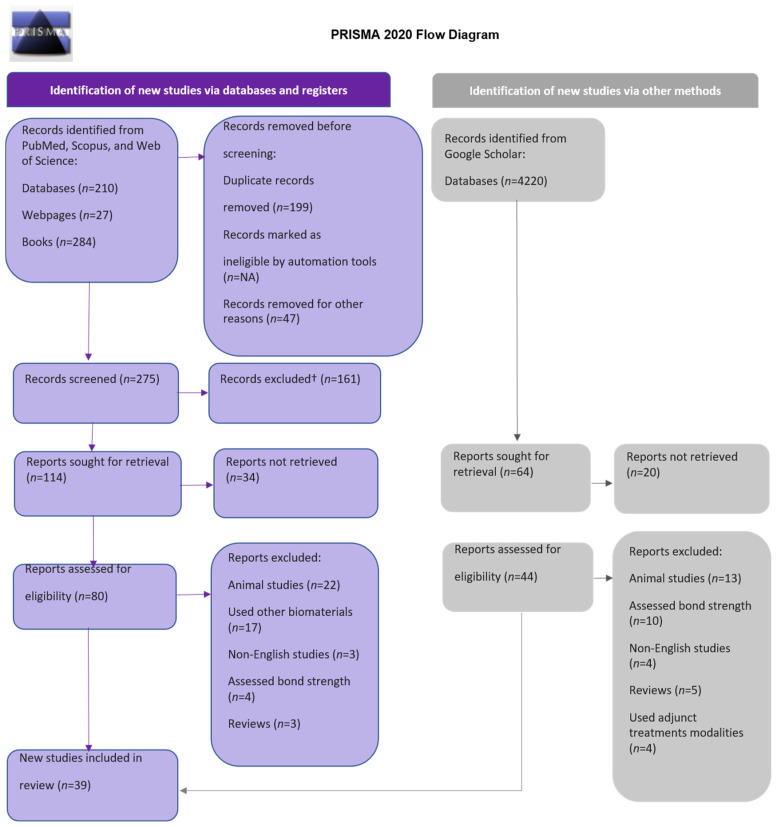
Flow diagram of PRISMA.

This systematic review is based only on original research articles carried out on human teeth. All the duplications were removed from the relevant articles using Mendeley^®^ (Elsevier Inc., Amsterdam, The Netherlands). In the analysis, only articles in English were used. The review process included a thorough assessment of abstracts of the articles for retaining the review quality and refinement in order to ensure the relevance and quality of the scholarly literature. Two independent reviewers then assessed the title and abstract of articles for relevance to the objectives of the study. Each pertinent study that matched the criteria was then screened for full-text content and then carefully evaluated. When there was a doubt, a consensus was made between the reviewers to exclude or include a study. Finally, after eliminating duplicate records, applying inclusion and exclusion criteria, removing unretrievable studies, and reaching a consensus among the reviewers, an aggregate of 124 articles were examined for eligibility. Finally, 39 publications were chosen for assessment and data analysis. The steps at every stage of the review process using the PRISMA 2020 flow diagram are shown in Figure 1.

### 2.4. Extraction of Data

In the stage of data extraction, thirty-nine articles only in the English language were selected, and characteristic findings were extracted from original research and case series articles from the field of Dentistry/Biomaterials/Chemistry.

Risk of Bias (RoB) categorization was determined by the number of conditions that were met using RevMan Version.5.3 software (The Cochrane Collaboration, London, UK). The criteria assessed were a selection bias in randomization and group allocation, a blinding of personnel and outcome assessment, and lastly a reporting bias. When all the requirements were met, the risk was deemed to be ‘low’, ‘moderate’ if there was one missing, and ‘high’ if there were more than two criteria missing [24].

### 2.5. Meta-Analysis

The analyses were based on seven studies. The extent of remineralization subgroup analysis included four articles, whereas the elastic modulus was analyzed using three articles. The effect size index is the standardized difference in means. The effect on either side of zero represents it if it favors the group treated with biomimetic agent(s) or the control. Because there were few papers with comparable criteria for evaluating study outcomes that were accessible, the fixed-effects model was used in the analysis. The estimated effect size was analyzed using the remineralization success ratio and the odds ratio with a 95% confidence interval. The existence of heterogeneity between the combined studies was assessed using the Q test (*p* < 0.05) and quantified with the I^2^ statistical index proposed by Higgins, which describes the percentage of the total variation between the studies due to heterogeneity rather than being random, quantifying the effect of heterogeneity between 0 and 100%; 25–50% was considered mild, 50–75% was considered moderate, and >75% was considered high. The Q intergroups test (*p* < 0.05) was used to assess the existence of differences in the success rate between the subgroups. Meta-analyses were represented by forest plots [25].

## 3. Results

### 3.1. Studies’ Allocation

The initial search of articles yielded 4741 results. The following number of articles were found in each of the databases: PubMed, 140; Scopus, 329; Web of Science, 21; grey literature source, 4220. After duplicate checks using Mendeley^®^ reference manager, the total records sought for retrieval were 178, from which 124 studies were checked for eligibility criteria. After applying inclusion and exclusion criteria, 75 studies were excluded for being animal studies, having used non-biomimetic agents, being non-English articles, being reviews, using adjunct treatment modalities, having assessed enamel hypoplastic lesion remineralization, and being resin–dentine interface studies such as bond strength assessment. According to the PRISMA 2020 flow diagram for systematic review, the results are displayed in Figure 1. Two authors defined and evaluated a data extraction protocol. One author extracted data from full-text articles, which were then reviewed by another author. The functions of the remineralizing agents and mode of action mentioned in the included articles were summarized.

### 3.2. Descriptive Analysis

Thirty-nine full-text research articles [19,26,27,28,29,30,31,32,33,34,35,36,37,38,39,40,41,42,43,44,45,46,47,48,49,50,51,52,53,54,55,56,57,58,59,60,61,62,63] were finalized for analyses. These articles were divided into two broad categories, viz., NCPs and Natural products, while only one study assessed amelogenin peptide for dentine remineralization. Table 1 shows characteristics of each article. Each of the included articles evaluated the human dentinal disc outcome measure. The likely confounding factors in the thirty-three incorporated studies could be attributed to variations in the thickness of each disc, the surface treatment prior to application of the remineralizing agent, the duration of remineralization, and different laboratory tests to determine the extent of remineralization and/or physical properties of the treated dentine.

### 3.3. Risk of Bias

**Figure 2:**
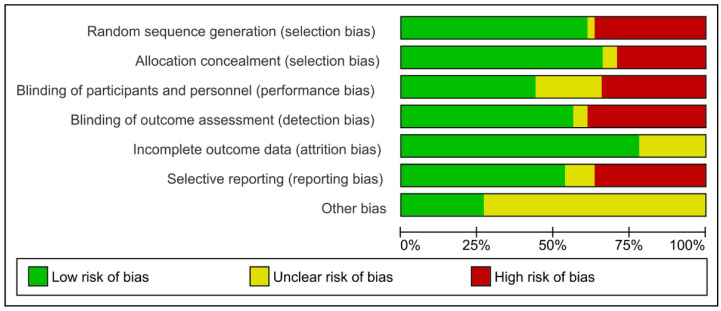
Risk of Bias.

The studies that were included had a high ROB of ~35% in blinding the personnel and individual participants to the outcome assessment. Allocation concealment and randomization had a low ROB of ~65%. The majority of the studies, ~75%, had unclear, other biases (Figure 2). Conclusively, a moderate quality of evidence was found, indicating that the findings could be satisfactory.

Out of the thirty-nine studies, four studies were in vivo RCTs, while 35 were in vitro. In vivo studies mainly assessed dentinal hypersensitivity treatment using hydroxyapatite. Post-treatment patients were assessed based on the visual analog scale [55,57], Airblast test [54], and Schiff sensitivity scale [53]. The occlusion of dentinal tubules was one of the criteria considered for the efficacy of desensitization of dentine.

Most of the thirty-five in vitro studies used extracted molar teeth except studies [32,37,40,46] that used premolar teeth. Each dentine slice’s thickness ranged from 1 to 4 mm. The surface treatment of dentine was performed to achieve a demineralized layer before biomimetic remineralization. Artificial lesion preparation offers advantages over natural lesions as they are reproducible. A lesion depth of 140 microns provides sufficient depth for the evaluation of remineralization [48]. The preparation of artificial carious lesions by the use of demineralizing solution either alone [37,48] or with remineralizing solution (pH-cycling procedure) [27,32,34,36,49,59] was reported. Some studies used 20–37% phosphoric acid to induce demineralization [28,29,30,38,40,43,47,51,58,61,63], followed by phosphorylation using STMP [58] or covered with a Portland-cement-based lining composite [61]. Some studies used 14–17% EDTA to induce demineralization [33,35,39,42,44,45,46,50,60], while other studies used acetate buffer solution [19,41]. One study used formic acid to completely demineralize dentine [62], and Mukherjee et al. used demineralizing buffer [31] followed by artificial saliva immersion. Calcium and phosphate ions were provided by a variety of remineralizing mediums. The most widely used of them was Portland cement, which served as a source of both calcium and hydroxyl ions.

The most commonly used NCP agents either alone or in combination were PAA [26,27,32,34,36,38,39,40,50,51,59,61,62,63] and STMP [26,27,30,36,42,58,61,63]. Other agents used were poly-amidoamine (PAMAM) [29,39,45,46] and aspartic acid [19,44,48].

Functions of PAA include simulating calcium phosphate binding sites of Dentine Matrix Protein-1 (DMP1). It serves as a sequestration agent to stabilize amorphous calcium phosphate before it enters dentine collagen fibrils and prevents fluidic ACP nanoparticles from aggregating into larger particles and changing into apatite. Furthermore, as the template-biomimetic-analog, a polyphosphate-containing agent such as PVPA or STMP was found to bond with the matrix of dentine collagen and draw ACP nanoprecursors into the collagen matrix. PVPA also functions as a DMP’s template-analog and simulates DMP1’s collagen-binding function, thereby inhibiting matrix metalloproteinases and attracting ACP nanoprecursors. It also phosphorylates type I collagen causing adsorption and covalent bond formation with the demineralized collagen matrix. It also acts as a template molecule, attracting ACP-nanoprecursors in nucleating the collagen fibrils [51,64,65]. These NCP analogs can attach to the collagen matrix, causing ACP nanoprecursors to be induced. It has been stated that polydopamine can interact with collagen by cross-linking and assisting in immobilizing NCPs onto dentine collagen fibers [28].

A phosphate-based glass powder was prepared as remineralizing medium with PAA [26], whereas artificial saliva was used in some studies [31,37,40,66]. Calcium chloride [38,41,42] was also used during the polymer-induced liquid precursor (PILP) process [19,39,48,59,60]. Metastable calcium phosphate remineralizing solution was used by Cao et al. [58]. Some studies used Portland cement [30,35,51,61,62,63], while others used simulated body fluid (SBF) [27,32,34,35,36,47]. One study used nanoparticles of amorphous calcium phosphate as a source of Ca/P ions [29], while another study used calcium and phosphate buffer in the presence of a 1 mA direct current for a constant flow of Ca_2_ and PO_4_ ions [33]. Potassium dihydrogen phosphate was used as a phosphate ion supplier [41,42].

The most commonly used tests to assess the efficacy of NCPs on dentine are scanning electron microscope/energy-dispersive X-ray absorptiometry (SEM/EDX), followed by Fourier transformation infrared spectroscopy (FTIR), transmission electron microscopy (TEM), X-ray diffraction (XRD), and micro-computed tomography (micro-CT). Micro-CT was found to have a high spatial resolution in 3D, showing a strong linear correlation between CT density and mineral content [67]. Table 2 shows the most commonly used assessment outcomes and their respective findings.

A study by Abou Neel et al. [20] found that the lumen of tubules was occluded completely after eight weeks of remineralization therapy. The fluid movement within the tubules could be reduced by this occlusion, thereby reducing dentinal hypersensitivity. Another study found out that the deposit showed a staggered arrangement similar to brushite and the remineralized zone was strongly adhered to the underlying dentinal layer [26]. Agglomerated spherical precipitates were observed when nanohydroxyapatite with NCP analogs were used to obliterate patents’ dentinal tubules [32].

In addition to different physical and biological properties of the remineralized dentine, as shown in Table 2, the main findings observed by the authors include:

1. Collagen fibrils can induce apatite nucleation and mineral deposition, but it is slow. Hence, additional nucleation templates are applied, and high concentrations of calcium and phosphorus ions are supplied [51,68,69,70]. Type 1 collagen allows molecules, peptides < 6 kDa, to diffuse freely into collagen compartments that are water-filled, thereby initiating HAP crystal nucleation [71]. Hence, NCPs, precursor mineral phase size exclusion, and polyelectrolyte-directed mineralization systems can work [72,73,74,75].
2. DMP1 is an acidic NCP [76,77] and formed by odontoblasts dispersed within the interior tubules and between collagen fibrils [78]. It has high glutamine and aspartic acid and, hence, has a high capacity to bind calcium ions. It serves two roles: 1. to inhibit the growth of individual crystals and also stabilize nanoclusters to prevent the further growth of calcium phosphate nuclei; 2. to promote controlled mineral nucleation when applied on self-assembled collagen templates [35]. NCP analogs can replicate the functional properties of real proteins because of their strong affinity for calcium and collagen [79].
3. The two most used NCPA analogs are PAA and STMP.
  PAA is a sequestration agent and stabilizes ions to form liquid-like nanoparticles. This helps to penetrate the water compartment of collagen.
  Matrix phosphoproteins’ collagen-binding activity is mimicked by STMP. It causes phosphate attachment on collagen to draw calcium ions and direct apatite crystal nucleation within the gap zone of collagen fibers [65]. When spaces between collagen become supersaturated, calcium and phosphorus precipitation occurs, causing stable hydroxyapatite formation [26]. Interfibrillar mineralization, which is caused by the precipitated particles being distributed uniformly and consistently along collagen fibers, gives the appearance of corn on the cob [51].
4. Other agents used are PAMAM, proanthocyanidins, aspartic acid, carboxy methyl chitosan, glutamic acid, grape seed extract, and agarose hydrogel. Some studies loaded PAMAM with triclosan or epigenin to increase its antibacterial efficacy [45,46]. (Refer Table 1).
5. Collagen fibrils and mineral phases around and within the collagen fibrils contribute to the total strength of dentine [80] in the form of interfibrillar mineral concentration [81] and intrafibrillar collagen mineralization [82]. Mechanical properties of dentine such as microhardness and elastic modulus are linearly related to mineral content [82]. The microhardness increases due to increasing extra fibrillar mineral concentration and volume, which consolidates the granular matrix [50]. Remineralized dentine collagen has a stronger resistance to degradation, thus impacting secondary caries [59].
6. ACP has also been used before, which is a solid-phase precipitate formed from super-saturated calcium phosphate solution. It has excellent bioactivity and high cell adhesion [60].
7. The collagen mineralization process is a bottom-up approach [60] centered on the non-classical theory of crystallization [65].
8. Calcium and phosphorus contents show a synchronous increase from superficial to deeper layers [59].

### 3.4. Meta-Analysis Results

**Figure 3:**
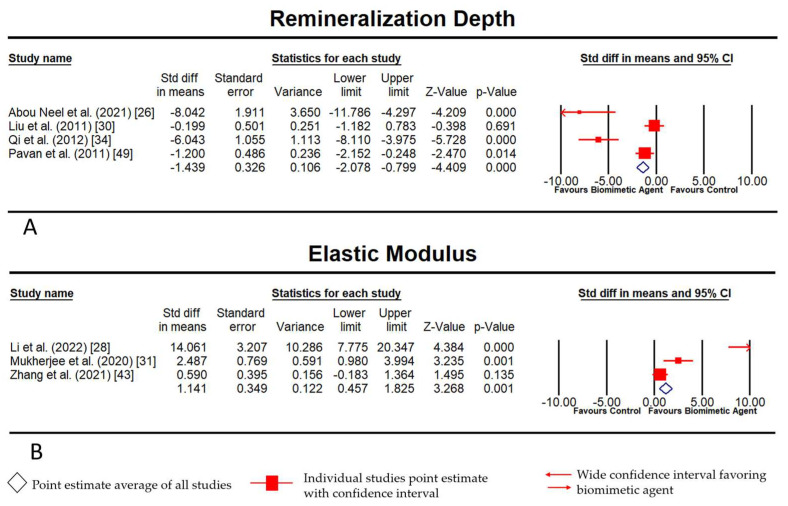
(**A**) Fixed-Model Meta-analysis for extent (depth) of remineralization; (**B**) Fixed-Model Meta-Analysis for elastic modulus physical characteristic of remineralized layer.

Comprehensive Meta Analysis^®^, v3.0, was used to generate the forest plot with a fixed-effects model of incorporated studies assessing two outcomes, viz., the extent of remineralization (Figure 3A) that showed a significant *p* value of 0.00, whereas the *p* value for the elastic modulus was 0.001 (Figure 3B), indicating that we can discard the null hypothesis. Some of the dispersion was caused by actual variations in the research effects rather than just random errors. In addition, it was observed that there was a large dispersion between the studies as shown in Figure 3. It, however, shows that the results favor biomimetic treatment more than we can attribute to chance. The Z value was 3.268 with *p* < 0.01 and, using a criterion of alpha at 0.05, we discarded the null hypothesis and concluded that the mean effect size was not precisely 0. The Q-statistic of 21.24 and *p* value of 0.00 prove that the size varied between studies. A high I^2^ statistic was observed in both outcomes (Extent of remineralization—90.56 and elastic modulus—91.96), suggesting that the reflection of proportion was due to true effects rather than sampling errors. Thus, it can be inferred from the final data that differences in methodology, treatment time, and concentration of the biomimetic agents in each group resulted in a significant heterogeneity amongst the studies.

## 4. Discussion

Biomineralization is a particle-based process through organic matrix proteins causing remineralization by apatite nucleation and growth [83]. Recently, a new treatment approach called ‘biomimetic remineralization’ has gained popularity as it has the capability to mimic the natural process of remineralization of dentine. Although the collagen matrix is widely known to serve as a scaffold for crystal deposition, it lacks a mechanism for the nucleation of hydroxyapatite [84].

A series of NCPs typically modulate the process of biomimetic mineralization, despite accounting for only about 10% of the organic components [58,85]. NCPs such as DMP1 and dentine phosphophoryn (DMP2, DPP) possess highly phosphorylated threonine and serine residues. They have a strong affinity for calcium ions and collagen fibrils, which controls mineral crystal nucleation and growth [86]. Although DMPs promote the deposition and nucleation of hydroxyapatite, there is no conclusive evidence that suggests anatomical and functional regeneration of the dentine [37]. As a result, studying the structures and functions of other synthetically modified NCP analogs becomes a general strategy for biomimetic remineralization of dentine’s sophisticated hierarchical structure.

As natural NCPs are difficult to extract and purify, several researchers are trying to develop their analogs. The most commonly used NCP analogs were PAA and STMP. Metastable ACP nanoprecursors become stabilized when PAA is added to the Portland-cement–SBF combination, and their size becomes small enough to permeate into a demineralized collagen matrix [30,51,63]. Polyvinyl phosphonic acid has also been used along with PAA, which mimics DMP1’s collagen-binding function to guide the ACP nanoprecursor to the collagen matrix [51].

Recent studies have also utilized a dual analog system to achieve better physical and biological properties of the remineralized dentine [26,27,28,30,32,34,38,39,40,51,63]. PAA has been used with a variety of biomineralization proteins such as L glutamic acid, which promotes crystallization kinetics, thus shortening the remineralization duration to two days [38]. Wang et al. used 1% sodium fluorescein with PAA and self-etch adhesive and demonstrated that the fluorescent mineralizing adhesive was non-toxic and its effect lasted for over six months [40]. Li et al. added polydopamine and found that the remineralized dentine had similar physical characteristics and acid resistance to that of enamel [28]. Phosphate-based template-analogs such as STMP or PVPA have also been combined with an ACP stabilization analog such as PAA, resulting in substantial variations in the depth of the lesion and the relative mineral composition of the lesion surface [30]. STMP can bind to type I collagen through covalent bonding, chemical phosphorylation, and electrostatic mechanisms [42,63,87].

The dentine collagen matrix, which is phosphorylated, resulted in both intrafibrillar as well as interfibrillar remineralization by acting as a template-molecule in attracting ACP nanoprecursors as well as nucleating apatite inside the collagen fibrils [30]. A study by Cao et al. in 2013 compared and assessed the effects of the non-phosphorylated and phosphorylated dentine collagen matrix on the intrafibrillar remineralization of dentine. They came to the conclusion that in the presence of ACP nanoprecursors, the non-phosphorylated dentine collagen matrix could not induce intrafibrillar remineralization [58]. In addition to the abovementioned biomimetic agents, phosphorylated chitosan [41], peptide/oligopeptide [88], and PAMAM dendrimer [45,89,90] further acted as template analogs for the remineralization of the dentine collagen matrix.

The integrity of the collagen matrix is vital for proper remineralization [91] to prevent H+ ions from penetrating the porous dentine and causing severe mineral loss (ΔZ) [91,92]. Type I collagen accounts for over 85% of the organic phase of dentine [93]. When dentine is demineralized, the acid produced by bacteria exposes type I collagen. Enzymes such as matrix metalloproteinases (MMPs) released during the natural dental caries process destroys the exposed collagen fibrils [94]. Type I collagen acts as templates for attracting ACP nanoprecursors. In vitro studies have utilized a variety of agents to expose type I collagen. Most commonly, 37% phosphoric acid was used, which does not denature the matrix of dentine collagen [95,96]. Phosphoric acid at various concentrations (20–37%) was utilized to reveal the dentine collagen matrix [1,45,47,58,88,90]. A five-micrometer-thick layer of a mineral-free collagen matrix was formed in just 15 s [51] and a 2–4 m-thick layer was formed with a 10 s exposure [97]. Other researchers obtained a 3–4 mm thick artificial demineralized dentine layer with 35% PA for 10 s [38] and a 5–8 mm-thick layer by etching with 32% phosphoric acid for 15 s [61]. The EDTA-etching method was also used to remove the mineral content. It could keep the dentine collagen matrix intact while also providing a mineral-free layer in close proximity to the dentine surface [89]. Additionally, the pH-cycling procedure was employed by many authors to produce partially demineralized dentine. It may be able to replicate the dynamic changes in mineral saturation that take place during the course of the natural caries process [27,30,32,34,36,37,49,59,98].

Liu et al. in 2011 [65] stated that the mechanisms present in the classical top-down approach depend on the epitaxial growth of seed crystallites inside the collagen fibrils, whereas the non-classical bottom-up mineralization approach includes changing ACP nanoparticles into apatite crystallites when biomimetic analogs are present. Metastable phosphate and calcium-ion-containing solutions as well as gels are frequently used in traditional ion-based mineralization strategies [99]. Minerals on the organic matrix cannot spontaneously form crystals, in order to achieve this top-down mineralization. In addition, this mechanism does not show intrafibrillar apatite deposition without the use of NCPs [60]. Some of the outcomes in this review demonstrated only the deposition of interfibrillar apatite and dentinal tubule occlusion when agents such as bioactive materials [100,101,102], an agarose gel system [47], and zinc [103] were used. Despite the fact that Wang et al. successfully synthesized peptide [88] to partially mimic the role of NCPs, they were unable to form ACP nanoparticle and, therefore, could not replicate the structural hierarchy of intrafibrillar apatite accumulation inside the collagen matrix. Nonetheless, these findings have clinical implications for treating dentine hypersensitivity.

The inorganic hydroxyapatite crystals can either be intrafibrillar crystallites that are oriented along the c-axis running parallel to the collagen fibrils or interfibrillar crystallites deposited between the collagen fibers [82,104]. Dentine’s interfibrillar mineral is rapidly dissolved after demineralization, whereas the intrafibrillar mineral is either partially or completely dissolved, revealing the gap zones. Its mechanical properties are greatly influenced by intrafibrillar mineral [105]. In order to cause intrafibrillar collagen mineralization, hydroxyapatite nucleation and growth are initiated and controlled by NCPs [86].

Another non-classical theory of crystallization also called a bottom-up approach, which begins with one or more distinct molecular class, goes through certain transformations resulting in an organized structure of amorphous nanoprecursor particles and their further mesoscopic alterations, ultimately resulting in the formation of a biomimetically induced remineralized layer [106]. This particle-mediated crystallization pathway was described by Dey et al. in 2010 as a multistage [107]. Prenucleation clusters form when phosphate and calcium ions self-assemble and aggregate into amorphous ACP nanoprecursors in the existence of NCP analogs. The precursors seep into gap zones of collagen fibrils and result in intrafibrillar remineralization. This also causes the interfibrillar remineralization of collagen fibrils [108].

With dental caries being a microbial-driven pathological process, some in vitro studies have added antibacterial agents along with different biomimetic agents to assess the activity of anti-caries against *S. mutans* [45,46]. Aciduric bacteria and their metabolic products are found abundantly within the infected dentine, making remineralization of the deeper layers difficult [46]. Hence, this modification may prove to be a breakthrough for developing delivery systems utilizing the anti-bacterial action of ACP-stabilization analogs.

In vivo studies [53,54,55,57] assessed nanohydroxyapatite in the treatment of dentinal hypersensitivity. All the studies have shown promising results. However, as pain is a subjective criterion, it could be a limitation factor in the assessment outcome.

Most of the studies on biomimetic remineralization have demonstrated the potential to remineralize carious dentine in a simplistic approach. However, as they were conducted in vitro, they have remained as a proof-of-concept and, thus, their use in clinical settings remains unexplored. Some studies have reported findings of an in vivo approach to study the effects of MTA application [109], of commercially available Biodentine^®^ [110], or in animals using a hydrogel system [111] on caries-affected dentine and showed varied results. It is, thus, pertinent to investigate the biomimetic remineralization procedure’s applicability to dentine carious lesions in vivo. Pellicle and biofilm formation still remain potential confounders that can impede the remineralization process. Considering these challenges, present reported strategies still have a long way to go before they can be employed in clinical care for the benefit of the patients.

Non-caries tooth loss due to non-bacterial acid attack results in the loss of superficial enamel and the exposure of underlying dentinal tubules. PAMAM has been successfully employed in tubule occlusion, thus reducing dentinal hypersensitivity [39].

The included studies had a common limitation in that it lacked information on the effect of the actual oral environment in the presence of oral biofluids and a plethora of microorganisms. Another critical aspect is toothbrushing under real conditions, which includes its effects on mechanical and chemical wear. In addition, the absence of the natural process of development of dental caries in the caries model poses yet another limitation. Although the results obtained from the experiments were favorable, they were carried out only in a controlled environment. Moreover, in vitro studies on dentine discs do lack the effect of pulp fluid as well. Hence, this necessitates addressing the above limitations in future studies.

This review thus attempts to fill the research gap that exists on the availability of newer techniques and methods of assessment of functional dentinal remineralization. It presents comparative data of the efficacy of various biomimetic agents and paves the way for future research. Due to the non-standardized approaches adopted by the studies, this review does not aim to provide concrete evidence of standard protocols in remineralization that can be universally accepted.

## 5. Perspective and Conclusions

The included studies assessing NCP and other naturally derived biomimetic agents showed effects with high heterogeneity and a large dispersion of results. Despite that, most of the studies stated an in vitro accomplishment in the biomimetic remineralization of dentine using various approaches, which includes the use of NCP analogs as well as biomaterials derived from natural sources. The first stage of biomimetic dentine remineralization is the creation of amorphous ACP nanoprecursors. A variety of NCP analogs were successful in interfibrillar and intrafibrillar remineralization of the demineralized dentine matrix. Biological effects also showed promising results even in the presence of bacterial load. However, these are in vitro models, which differ from in vivo conditions. Conclusively, it is pertinent to develop a universal method to assess biomimetic remineralization.

## Figures and Tables

**Table 1:** Full-text articles included for review of efficacy of biomimetic agent(s) in dentin remineralization.

S. No.	Biomimetic Remineralizing Agent	Study Type	Type of Agent *	Teeth Used	Sample Size	Reference
1.	Sodium Trimetaphosphate (STMP) and Polyacrylic Acid (PAA)	In vitro	1	Molars	4	[26]
2.	Dentine Matrix Proteins (DMP1) derivatives	In vitro	1	Premolars	12	[37]
3.	Poly-L-Aspartic Acid	In vitro	1	Third molars	-	[48]
4.	0.2 M STMP	In vitro	1	Third molars	2	[58]
5.	Carboxymethyl Chitosan (CMC)	In vitro	2	Third molars	10	[60]
6.	PAA, Poly-aspartic Acid (PASP)	In vitro	1	Third molars	10	[59]
7.	Poly L-Aspartic Acid	In vitro	1	Third molars	6	[19]
8.	PAA, Polyvinylphosphonic Acid (PVPA)	In vitro	1	Third molars	10	[62]
9.	STMP and PAA	In vitro	1	Third molars	10	[63]
10.	STMP And PAA	In vitro	1	Third molars	6	[61]
11.	3% PAA, 8% STMP	In vitro	1	Third molars	18	[27]
12.	PAA	In vitro	1	Third molars	20	[28]
13.	G3-PAMAM-COOH With Amorphous Calcium Phosphate	In vitro	1	Third molars	6	[29]
14.	STMP	In vitro	1	Third molars	8	[30]
15.	Peptide P26	In vitro	3	Third molars	6	[31]
16.	PAA, Sodium Tri-Poly Phosphate (STPP)	In vitro	1	Premolars	10	[32]
17.	Agarose Hydrogel	In vitro	2	Third molars	-	[47]
18.	DMP1-Derived Peptides Pa and Pb	In vitro	1	Third molars	-	[33]
19.	Grape Seed Extract Powder (Active ingredient Proanthocyanidin)	In vitro	2	Third molars	10	[49]
20.	PAA and Sodium Tri-Poly Phosphate (STPP)	In vitro	1	Third molars	10	[34]
21.	G4-PAMAM-PO_3_H_2_ With PAMAM-COOH	In vitro	1	Third molars	-	[35]
22.	PAA and STMP	In vitro	1	Third molars	60	[36]
23.	PAA and L-Glutamic Acid (L-Glu)	In vitro	1	Third molars	-	[38]
24.	PAA and PVPA	In vitro	1	Third molars	20	[51]
25.	Phosphorylated PAMAM Dendrimers with PAA	In vitro	1	Third molars	16	[39]
26.	1 wt% of Sodium Fluorescein And 25 Wt% of PAA with ACP	In vitro	1	-	20	[40]
27.	Mollusk-shell-derived Ca^2+^-Mg^2+^ with PAA	In vitro	2	Third molars	10	[50]
28.	P-Chitosan (Coated or Crosslinked)	In vitro	2	Third molars	6	[41]
29.	STMP	In vitro	1	Third molars	6	[42]
30.	Proanthocyanidin and Carboxymethyl Chitosan	In vitro	2	Third molars	20	[43]
31.	DL-Aspartic Amino Acid	In vitro	1	Third molars	10	[44]
32.	Triclosan-Loaded PAMAM Dendrimer At 3000, 6000 ppm Concentration	In vitro	1	Third molars	12	[45]
33.	Epigenin-Loaded PAMAM Dendrimer	In vitro	1	Premolars	12	[46]
34.	Zinc Hydroxyapatite	In vitro	4	Third molars	8	[52]
35.	Zinc Carbonate Hydroxyapatite	In vivo RCT **	4	Incisors, canine, premolar	72	[53]
36.	Zinc Carbonate Hydroxyapatite	In vivo RCT **	4	-	36	[54]
37.	Zinc Carbonate Hydroxyapatite	In vivo RCT **	4	-	29	[55]
38.	Nano-Hydroxyapatite	In vitro	4	Third molars	25	[56]
39.	Zinc Hydroxyapatite	In vivo RCT **	4	-	48	[57]

* NCP Analog: 1; Natural source: 2; Amelogenin peptide: 3; Hydroxyapatite: 4. ** Randomized Controlled Trial.

**Table 2:** Physical and biological properties of remineralized dentine.

Study Outcome	Findings	References
Extent of remineralization/Remineralization efficacy	90 to 98% remineralization to pre-treatment levels The highest remineralization of 0.9 mm after 4 months was observed by Gu et al. [61] The least remineralization reported was 5 µm by Wang et al. [39]	[26,27,29,30,34,37,39,40,44,48,49,50,51,59,60,61]
Elastic modulus	Li et al. reported the highest elastic modulus of 88.78 ± 8.35 GPa [28], whereas Ye et al. had the lowest of 22.17 ± 3.54 [43].	[19,28,31,43]
Ca/P content	P26 peptide showed the highest Ca/P ratio of 1.87 ± 0.014 and DMP-derived peptides A and B had the lowest of 1.58 ± 0.23	[29,31,32,33,35,39,45,46]
Hardness/Microhardness	0.37 to 0.65 GPa of hardness was observed and 43 to 95 HVN of microhardness was observed.	[28,29,31,32,36,38,43,50]
Cytocompatibility/cytotoxicity/Cell viability	Antibacterial effect against *S. mutans* when apigenin was used. Favorable biocompatibility. Cell viability up to 60–110% with triclosan.	[28,39,40,45,46,50]
Antibacterial	Zhang et al. showed *S. mutans* reduction by 97.3% and *L. acidophilus* by 95.7% [43]. Nambiar et al. observed a 1.5 mm zone of inhibition [32].	[32,43,46]
Dentine permeability	Up to 41% reduction.	[44,50]
Acid resistance	Liang et al. observed an increase in pH to 6.9 from 4.0 after 2 weeks of treatment [29].	[28,29]
Crystal size	1–3 µm.	[33,41,47]
Integrated mineral loss (DZ)	Surface was heavily remineralized than deeper portion. Significant reduction in mineral loss after 6 weeks of treatment.	[34,49]
Young’s modulus	Intrafibrillar-mineral-related Young’s modulus recovered in a short time due to Glu-controlled cooperative effect, giving flakelike crystallite formation.	[38]
Flexural modulus	58–118% increase.	[62]
Mineral density gain in %	6.83–14.43%.	[31]
Tensile strength	34.4 ± 19.94 MPa.	[31]
Friction coefficient and wear depth	0.12 ± 0.02 friction coefficient; wear depth observed was 5.3 ± 3.37 mm.	[28]
Occluded dentinal tubules	Complete occlusion observed after 4 to 8 weeks and resisted acid challenge	[52,56]

## Data Availability

Not applicable.
